# “Super Sandwich” Assay Using Phenylboronic Acid for the Detection of *E. coli* Contamination: Methods for Application

**DOI:** 10.3390/microorganisms13122745

**Published:** 2025-12-02

**Authors:** Anna N. Berlina, Svetlana I. Kasatkina, Margarita O. Shleeva, Anatoly V. Zherdev, Boris B. Dzantiev

**Affiliations:** A.N. Bach Institute of Biochemistry, Research Center of Biotechnology of the Russian Academy of Sciences, 119071 Moscow, Russia; kasatkinasvetlana7722@gmail.com (S.I.K.); mshleeva@inbi.ras.ru (M.O.S.); zherdev@inbi.ras.ru (A.V.Z.); dzantiev@inbi.ras.ru (B.B.D.)

**Keywords:** cell wall, lipopolysaccharide fragment, boronic group, ELSA, bacterial strain

## Abstract

This paper proposes a method for *E. coli* detection in a microplate format using low-molecular-weight compounds that specifically interact with the lipopolysaccharides (LPSs) of *E. coli* cell walls. These compounds can amplify analytical signals by binding to multiple repeating cell surface structures, while the selectivity for *E. coli* is ensured by preliminary cultivation on selective media, such as Endo or MacConkey agar. 3-Aminophenylboronic acid (APBA) was selected as the binding reagent for detecting *E. coli* LPSs. Conjugates of streptavidin (STP) and bovine serum albumin (BSA) with APBA and conjugates of biotin and soybean trypsin inhibitor (STI) and BSA were synthesized. The conditions for the sequential formation of “sandwich” type complexes (BSA-APBA conjugate/*E. coli*/STP-APBA/STI–biotin/STP–peroxidase) and their colorimetric detection using chromogenic peroxidase substrate were determined. The detection limit was 3 × 10^2^ cells/mL, and the range of quantitative determination covered five orders of magnitude—from 10^3^ to 10^8^ cells/mL. The developed assay was successfully tested using inactivated cells of pathogenic *E. coli* strains, confirming its potential for application. The assay was demonstrated to have universality, with the ability to detect *E. coli*, other bacterial pathogens, and LPS alone. This method could be adopted for the quantitative determination of different specific bacterial species using different selective media.

## 1. Introduction

*Escherichia coli* is a Gram-negative bacterium with a long history [1,2]. As a facultative anaerobe, this species can not only inhabit the lower intestines of mammals, but it can also survive in a variety of different environments [3,4,5]. Most strains are nonpathogenic or opportunistic, but the *E. coli* O157:H7 strain is highly virulent [1,6]. Pathogenic forms of E. coli cause various types of foodborne toxic infections that can result in severe damage to the human gastrointestinal tract and lead to sepsis, meningitis, uremic and hemolytic syndromes, etc. [7,8]. Therefore, water and food products should be monitored for the presence of these strains, along with the total coliform counts [9].

As a Gram-negative bacterium, *E. coli* has a cell wall that contains lipopolysaccharides (LPSs) on its surface, which is an important component of the outer membrane [10]. LPSs consist of three regions: lipid A, a core polysaccharide, and an outer fragment, the O-antigen, which determines the strain’s identity [10]. There are a huge number of *E. coli* serotypes, which have different virulence factors that determine the course of the disease when they infect a host [1,2,6].

Currently, microbiological [11], molecular genetic [12], and immunochemical [13,14] methods are used to detect pathogens such as *E. coli* in the environment and food, or for medical diagnostics, with each method having its own advantages and limitations [12,15,16]. Therefore, alternative solutions that provide easy testing and fast results are in demand. The search for and application of various molecules capable of interacting with the cell wall of *E. coli* is of interest for this task [17,18].

There are several directions that can be considered for developing analytical systems. These can be divided depending on the level of organization of the living matter that will be analyzed: the molecular, corpuscular, or cellular level.

The first approach is to target a fragment. For example, the authors of [19,20] demonstrated that the application of mannose to the surface of nanoparticles can positively influence the binding of *E. coli* cells. Mannose specifically binds to the pili protein on the cell surface. This mobile part of the cell contains various proteins, including FimH protein in flagella, which has a high affinity for mannose, as demonstrated in fundamental studies on this phenomenon [21,22,23,24].

If the LPS structure is considered not as a single fragment, but rather as monosaccharides containing various functional groups, then specific functional groups or local fragments of monosaccharides can be targeted [25,26]. For example, some lectins are capable of interacting with certain monosaccharides [27,28,29], and boric acid derivatives interact quite selectively with the cis-diol fragments of the monosaccharides in LPS [25,30,31,32,33].

The second approach is to detect whole cells, fragments, or individual molecules. The most sensitive methods are those that detect individual molecules; this is the basis for the molecular genetic tests that detect multiple copies of a pathogen’s DNA/RNA [12,34].

Antibodies can be used to recognize individual fragments of bacterial wall lipopolysaccharides. In this approach, immunization with whole cells results in the production of antibody clones capable of recognizing the molecular mixture on the LPS surface [35]. If antibodies are generated against a single element, for example, the O-antigen of *E. coli* LPS, they will only recognize and bind to cells containing the same fragment, i.e., cells belonging to the O-serogroup [36,37,38].

Endotoxins can be considered individual cell fragments, and various agglutination tests, including the well-known LAL test, have been developed to detect them [39,40]. Antigens on the surface of red blood cells specifically bind to fragments of bacterial LPSs (endotoxins), forming cross-links and resulting in visible red blood cell agglutination.

Finally, methods to identify whole living cells, specifically colony-forming units, include the microbiological method. In this method, bacteria are inoculated on the surface of solid nutrient media or within a liquid broth, followed by counting and identification of the microorganisms using complementary methods. A significant advance in microbiology was the development of selective nutrient media, the use of which significantly facilitates the process of microorganism identification [41,42,43].

**Figure 1:**
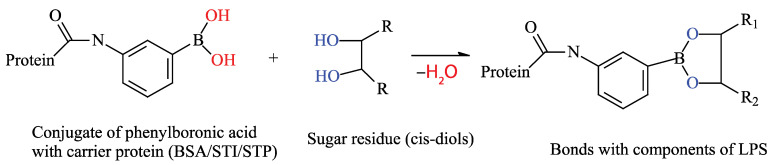
Interaction principle of the developed analytical system. The boron moiety of the acid reacts with the *cis*-diols of monosaccharides found in the O-antigen of the lipopolysaccharides of *E. coli* bacteria, forming a strong cyclic complex. If the BSA-APBA conjugate is used for immobilization, it will bind the bacterial CGCs to the wells of the plate. If APBA is used to bind immobilized cells, the complex can form in the wells of a microplate, which can then be detected.

The aim of this work was to develop an analytical system that combines interactions at the molecular and cellular levels. In this system, various media are used to obtain cell masses and individual colonies, and a boric acid derivative that specifically interacts with LPS fragments is used to detect *E. coli* at the molecular level (Figure 1).

However, there is a wide variety of Gram-negative bacteria, which form ecosystems that often also include *E. coli*, that also contain lipopolysaccharides on their cell walls. Therefore, the developed analytical system utilizes two approaches: a broadly selective approach that captures a variety of microorganisms, and a selective approach that specifically targets *E. coli*. The opportunistic *E. coli* ATCC 25922 strain was used to optimize the proposed scheme, including an assessment of the applicability of the selected selective media for cell mass growth, and then the system was validated using pathogenic strains of *E. coli* and other Gram-negative bacteria to demonstrate the advantages of the developed method.

## 2. Materials and Methods

### 2.1. Materials

3-aminophenylboronic acid (APBA), Tris, bovine serum albumin (BSA), soybean trypsin inhibitor (STI), dimethylsulfoxide (DMSO), N-(3-dimethylaminopropyl)-N′-ethylcarbodiimide hydrochloride (EDC), sulfo-N-hydroxysuccinimide (NHS), Triton X-100, biotinamidohexanoyl-6-aminohexanoic acid N-hydroxysuccinimide ester (biotin-NHS), and 2-morpholineethane sulfonic acid monohydrate (MES) were purchased from Sigma-Aldrich (St. Louis, MO, USA). Native recombinant streptavidin (STP) and STP conjugated with horseradish peroxidase (STP-HRP) were obtained from Imtek (Moscow, Russia). Pharmaceutical-grade gelatin powder (pure EP, NF) was purchased from Khimmed (Moscow, Russia). A ready-to-use 3,3′,5,5′-tetramethylbenzidine (TMB) peroxidase substrate with H_2_O_2_ (Immunotech, Moscow, Russia) was applied as the substrate for peroxidase.

Analytical-grade salts for buffers and all other reagents were purchased from Khimmed (Moscow, Russia). Deionized water with a resistance of 18.5 MΩ· cm at 22 °C was obtained using a Milli-Q Simplicity system from Millipore (Bedford, MA, USA) and was used to prepare all aqueous solutions. The following buffer solutions were used in this study: 50 mM Tris-HCl, pH 8.6 (Tris buffer); 50 mM phosphate buffer, pH 7.4 (PB); PB with 0.01% Triton X-100 (PBT-1); PB with 0.1% gelatin (PBG); 10 mM PB, pH 7.4 (PB-10); 50 mM carbonate buffer, pH 9.6 (carbonate buffer); 50 mM MES, pH 5.0 (MES); and PB-10 with 1% BSA (blocking buffer).

A Shaker IntelliMixer (ELMI, Riga, Latvia) was used for the conjugate synthesis. UV–vis spectra of the native proteins, compounds, and their conjugates were recorded using a Biochrom Libra S80 spectrophotometer (Biochrom Ltd., Cambridge, UK). A ZENYTH 3100 vertical photometer (Anthos Labtec Instruments, Wals, Austria) was used to detect the optical density in the microplate wells.

### 2.2. Methods

#### 2.2.1. Synthesis of APBA Conjugates with BSA and STP

Two methods for synthesizing conjugates with APBA were used. The first was implemented in Tris buffer, and the second in MES. The first method was performed using the protocol from our previous work [44] with modifications. The reactant solutions were prepared immediately before synthesis in amber glass flasks.

A 200 µL volume of an EDC water solution (290 mg/mL) was added to 1 mL of a BSA solution (10 mg/mL in 50 mM Tris buffer) and 0.5 mL of distilled water for carboxyl group activation. Then, 25, 50, 100, 200, or 300 μL of an APBA solution (20 mg/mL in DMSO) was added in a dropwise manner to synthesize BSA-APBA-0.5, BSA-APBA-1.0, BSA-APBA-2.0, BSA-APBA-4.0, and BSA-APBA-6.0 conjugates. Afterwards, the reaction mixture was incubated with continuous stirring for 1.5 h. The obtained conjugate was purified by dialysis against PB-10 overnight, and then stored as 50–100 µL aliquots at −20 °C.

For the STP conjugates, 2 mg of protein (200 µL of a 10 mg/mL solution in PB-10) was diluted in 1 mL of Tris buffer, and 200 µL of an EDC solution (30 mg/mL in water) and 25 µL of an APBA solution (20 mg/mL in DMSO) were sequentially added. After 1.5 h, the conjugate was dialyzed against PB-10, and then stored as 50–100 µL aliquots at −20 °C.

In the second method, two activators (EDC and sulfo-NHS) were used, as recommended in [45]. EDC and NHS water solutions (100 mg/mL, 300 µL each) were added to 1 mL of a BSA solution (10 mg/mL in 50 mM MES buffer) and 0.5 mL of distilled water for carboxyl group activation. Then, 100 μL of an APBA solution (40 mg/mL in DMSO) was added in a dropwise manner to synthesize BSA-APBA-4.0 (MES). Afterwards, the reaction mixture was incubated at RT with continuous stirring for 1.5 h.

For STP-APBA (MES), EDC and NHS water solutions (100 mg/mL, 30 µL each) were added to 1 mL of an STP solution (10 mg/mL in 50 mM MES buffer). Then, 15 μL of an APBA solution (40 mg/mL in DMSO) was added in a dropwise manner to synthesize STP-APBA (MES). The reaction mixture was incubated at RT with continuous stirring for 1.5 h. The obtained conjugates were purified by dialysis against PB-10 overnight, and then stored as 100 µL aliquots at −20 °C.

#### 2.2.2. Biotinylation of Proteins

STI and BSA proteins were mixed with biotin–NHS ester in a molar ratio of 1:65 for BSA and 1:15 for STI, and incubated for 2 h at room temperature with stirring. A 12.4 µL volume of the biotin–NHS solution (50 mg/mL) was added to 2 mg of BSA in 0.5 mL carbonate buffer. A 9.2 µL volume of the biotin–NHS solution (50 mg/mL) was added to 2 mg of STI in 0.5 mL of carbonate buffer and incubated for 2 h at RT. The reaction products were dialyzed against PB-10 using Amicon Ultracel 3K centrifuge filters (Millipore, Bedford, MA, USA).

#### 2.2.3. Determination of Free Amino Groups in the Synthesized Conjugates

Determination of free amino groups in the conjugates was carried out according to the method described in [46] with minor modifications. Briefly, 10 mg of fluorescamine (Sigma, St. Louis, MO, USA) was dissolved in 150 μL of acetonitrile in accordance with Fluram’s solubility. A 10 μL volume of the fluorescamine solution was added to 200 μL of each protein or conjugate solution (20 μg/mL in PB) and kept for 15 min at RT with shaking to obtain the labeled protein. After this reaction, 150 μL of each solution was transferred to the wells of white microplates (Nunc, Roskilde, Denmark) for fluorescence measurements (excitation wavelength of 390 nm in the range of 450–550 nm) using an EnSpire 2300 microplate reader (Perkin Elmer, Waltham, MA, USA). The absorbance at the emission maximum of 490 nm (from the spectra) was used to calculate the decrease in fluorescence for all pairs of proteins and their conjugates.

#### 2.2.4. Characterization of the Conjugates by FT-IR Spectroscopy

The synthesized conjugates were lyophilized using a Martin Christ freeze dryer (Alpha 1–2 LD Plus, Osterode, Germany). Fourier-transform infrared (FT-IR) spectroscopy was performed on all preparations, including native proteins (BSA and STP) and their conjugates with APBA (BSA-APBA and STP-APBA, synthesized under different conditions), and all lyophilized preparations. The spectra were obtained in the wavelength range between 4000 and 400 cm^−1^ using an FT/IR-6700 spectrophotometer (JASCO, Tokyo, Japan).

#### 2.2.5. Bacterial Strain and Cultivation Conditions in Cultural Media

The *Escherichia coli* ATCC 25922 strain, other Gram-negative bacteria, and *Staphylococcus aureus* ATCC 25923 (a Gram-positive bacterium) were obtained from the State Research Center for Applied Biotechnology and Microbiology, Obolensk, Russia. The *Mycobacterium smegmatis* mc^2^155 strain (ATCC 700084) was obtained from the All-Russian State Institute for Control and Standardization of Veterinary Preparations (Moscow, Russia). The inoculum was initially grown from a stock stored at −70 °C in 40% glycerol. The cells were cultivated for one night in 30–200 mL of meat-peptone broth (MPB) (Himedia, Thane, India) at 37 °C with stirring (200 rpm). Before inoculation, the meat-peptone broth and meat-peptone agar were autoclaved for 20 min at 121 °C.

Cell cultures were grown in two 20 mL flasks. The cultures were centrifuged for 15 min at 4500× *g* at 4 °C, and washed twice with sterile PB. The initial culture medium was discarded, and the cells were resuspended in sterile PB. The OD of the cell suspension was measured at 580 nm on the day of the experiment. The cultures were sown onto solid agar to determine the exact concentration of *E. coli* (in CFU/mL) after 24 h.

MacConkey and Endo selective media were prepared according to the instructions and autoclaved for 15 min at 121 °C. After cooling, they were poured into Petri dishes. To prepare Endo medium, the agar content was increased to 1.5% to ensure the required density for seeding the cell culture. This medium was autoclaved for 5 min at 100 °C. Fresh inocula (100 µL each) were placed onto the MacConkey and Endo selective media with agar and incubated for 18 h at 37 °C. The bacterial biomass was resuspended in PB-10.

#### 2.2.6. Microscopy of *E. coli* Cells

*E.coli* cells were analyzed using phase-contrast fluorescent microscopy. A 2 µL volume of a propidium iodide solution (0.1 mg/mL) was added to 100 µL of the cell suspension in an Eppendorf tube, and incubated for approximately 5 min. Then, 3 µL of the reaction mixture was applied to a glass slide and covered with a coverslip. The resulting preparations were analyzed using a Nikon fluorescence microscope (excitation at 510–560 nm and emission at 590 nm for propidium iodide). Photos were taken using a DS Qi2 camera (Nikon, Tokyo, Japan).

#### 2.2.7. Enzyme-Linked Sorbent Assay (ELSA) for *E. coli* ATCC 25922 Detection

BSA-APBA conjugates with different ratios (BSA-APBA-0.5, BSA-APBA-1.0, BSA-APBA-2.0, BSA-APBA-4.0, and BSA-APBA-6.0) at a concentration of 2.0 μg/mL in 10 mM PBS were adsorbed into the wells of a microplate overnight at 4 °C. Then, the microplate was washed once with 50 mM PBT containing 0.01% Triton X-100 (PBT-1) and twice with PB. Then, a suspension of *E. coli* ATCC 25922 cells was added to the wells at different concentrations (from 600 to 10^9^ cells/mL or from 10 to 10^8^ cells/mL in PB) and incubated for 90 min at 37 °C. After washing with PBT-1 and PB, 2 µg/mL STP-APBA conjugate in PBG was added. Then, a biotinylated protein conjugate (BSA–biotin or STI–biotin at 2 µg/mL in PBG) was added and the plate was incubated for 1 h at 37 °C. After washing with PBT-1 and PB, a 1:5000 solution of STP-HRP (Imtek) in PBS was added and the plate was incubated for 1 h at 37 °C. The activity of the enzymatic label bound to the carrier was determined by adding 100 μL of a substrate solution (commercial TMB + H_2_O_2_) and stopping the reaction after 15 min with 50 μL of 0.1 M H_2_SO_4_. Optical density was measured at 450 nm.

The optical density at 450 nm vs. the cell concentration or logarithm of *E. coli* cell concentration in the microplate wells was plotted and statistically analyzed using Origin 9.0 software (Northampton, MA, USA). The results were calculated using a 4 PL (4 Parameter Logistic) curve fit at https://www.aatbio.com/ (accessed on 19 November 2025).

#### 2.2.8. Pathogen Detection by ELSA

The BSA-APBA and BSA-APBA-4.0 conjugates (2.0 μg/mL in 10 mM PBS) were adsorbed into the wells of a microplate overnight at 4 °C. Then, the microplate was washed once with PBT-1 and twice with PB. A suspension of *E. coli* O157:H7 cells was added to the wells at different concentrations (from 100 to 10^8^ cells/mL in PB) and incubated for 90 min at 37 °C. After washing with PBT-1 and PB, 2 µg/mL of STP-APBA conjugate in PBG was added. Then, 2 µg/mL of STI–biotin conjugate in PBG was added and the plate was incubated for 1 h at 37 °C. After washing with PBT-1 and PB, a 1:5000 solution of STP-HRP (Imtek, Moscow, Russia) in PBS was added and the plate was incubated for 1 h at 37 °C. The activity of the enzymatic label bound to the carrier was determined by adding 100 μL of a substrate solution (commercial TMB + H_2_O_2_) and stopping the reaction after 3 min with 50 μL of 0.1 M H_2_SO_4_. Optical density was measured at 450 nm.

## 3. Results and Discussion

### 3.1. Proposed Scheme

**Figure 2:**
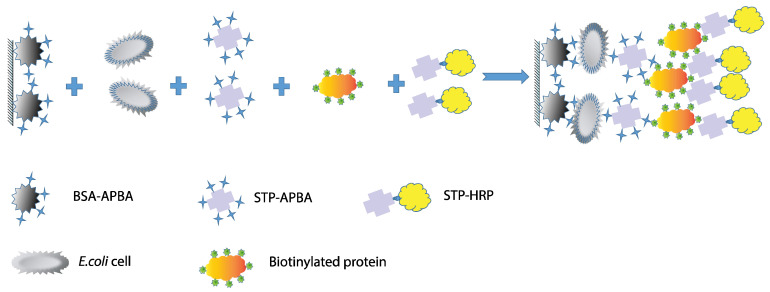
The proposed “sandwich” scheme for *E. coli* detection.

The presence of lipopolysaccharides on the cell walls of *E. coli*, a Gram-negative bacterium, enables the use of various types of multivalent interactions. The presence of multiple repeating carbohydrate backbones on the surface allows for the use of low-molecular-weight compounds to increase the number of complexes formed on the cell surface. Therefore, this study proposes a scheme involving a “sandwich” interaction using phenylboronic acid conjugates, which bind to *cis*-diols within the O-antigen on *E. coli* cells. The canonical structure of LPSs, specifically the O-antigen, suggests the presence of monosaccharides such as rhamnose, glucose, and galactose, as well as the presence of N-acetyl fragments of sugars. Therefore, the presence of *cis*-diol fragments in their structures can interact with a phenylboronic acid derivative [33] (Figure 2). Thus, in the proposed method, a BSA–aminophenylboronic acid conjugate is immobilized in the wells of a microplate. Cells are then introduced, followed by a streptavidin conjugate with the same boron derivative, a biotinylated protein, and then a streptavidin–peroxidase co-conjugate again. The biotinylated protein in this scheme acts as a “lock,” linking the two streptavidin conjugates with strong bonds. All conjugate preparations except for the commercial STP-HRP conjugate were synthesized for this work.

The advantage of this scheme is the use of conjugates obtained by covalently conjugating carrier proteins with molecules capable of binding to bacterial LPSs. The use of high-affinity streptavidin–biotin interactions facilitates the labeling of cell complexes and allows for their quantitative detection in microplate wells. This scheme eliminates the use of expensive antibodies whose cell binding depends on recognition specificity, as antibodies recognizing one strain typically do not interact with other strains. This detection scheme allows for cross-reactivity and utilizes the formation of strong chemical bonds.

### 3.2. Preparation and Characterization of Conjugates with Aminophenylboronic Acid

**Figure 3:**
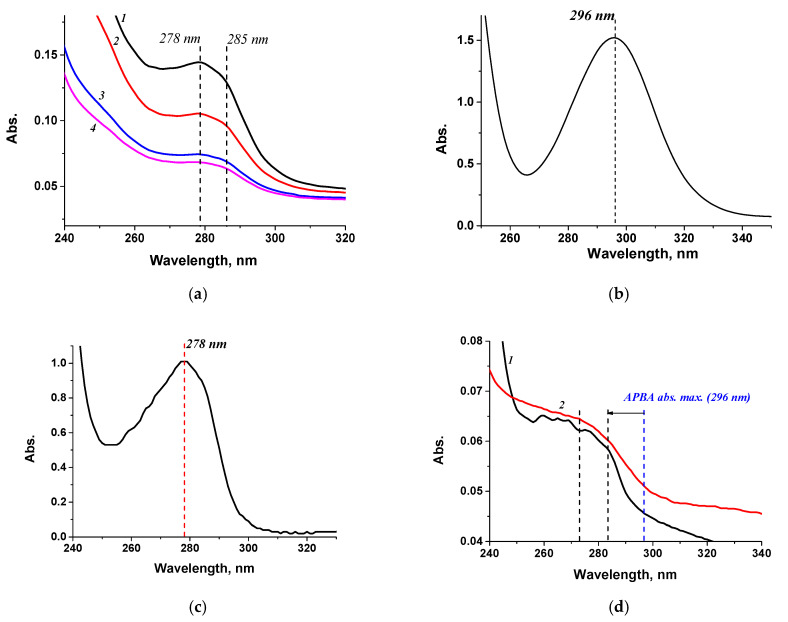
Absorption spectra of (**a**) BSA-APBA-1.0 (1 mg/mL) (*1*), BSA-APBA-2.0 (0.8 mg/mL) (*2*), BSA-APBA-4.0 (0.5 mg/mL) (*3*), BSA-APBA-6.0 (0.5 mg/mL) (*4*); (**b**) APBA (1 mg/mL); (**c**) BSA (1 mg/mL); (**d**) STP-APBA (*1*) and STP (*2*).

BSA-APBA preparations were synthesized using various protein-to-hapten ratios since the capabilities of this system were unclear. To obtain the STP-APBA conjugate, the ratio was not varied and was based on the number of carboxyl groups in the streptavidin structure (monoaminodicarboxylic amino acid residues) [47]. Each STP monomer contains eight of these residues, for a maximum of 32, without considering availability during tetramer formation [47]. All resulting conjugate preparations were characterized by spectrophotometry; the spectra are shown in Figure 3. As can be seen from the spectra, conjugate synthesis resulted in a leftward shift of the native APBA absorption peak at 296 nm, and the spectrum was flatter compared with the native components (the carrier protein and aminophenylboric acid) (Figure 3a–c). The spectrum also changed during the synthesis of the STP-APBA conjugate (Figure 3b,d). All obtained conjugates were analyzed during the optimization of interaction conditions.

In this study, biotinylated protein was chosen as the reagent that binds the two streptavidin conjugates. The high-affinity interaction between biotin and streptavidin results in a strongly bound complex with the peroxidase label. This interaction increases the sensitivity of the assay through sequential assembly of the complex. Why was a biotin–HRP or HRP-APBA conjugate not used? The conjugation process utilizes biotin’s N-hydroxysuccinimide ester with an activated carboxyl group. This means that conjugation with peroxidase requires the protein’s amino groups. However, horseradish peroxidase only has six amino groups [48], some of which are in the active site or hidden within the globule, making this conjugation impractical. Regarding the synthesis and use of the HRP-APBA conjugate, its production is complicated by the enzyme’s 18% glycosylation at asparagine residues in positions 13, 57, 158, 186, 198, 214, 255, and 268 [48], and the presenting sugar residues are able to interact with APBA. Therefore, obtaining such a conjugate is impossible and could be prone to non-reproducible results and difficulty in accessing the boron moiety.

All the synthesized conjugates were characterized. The free amino groups for all conjugate preparations were determined using fluorescamine. The fluorescence of conjugate solutions at 490 nm was compared to that of the original carrier proteins used in their synthesis. The number of amino groups for each protein was estimated based on amino acid sequence data from the PDB databases. The data obtained from fluorescence measurements of the labeled proteins are presented in Table 1. In the STI–biotin and BSA–biotin conjugates, the amino groups of the protein interact with the carboxyl groups of the biotin derivative. Based on the fluorescence data, the protein-to-hapten ratio for these preparations was calculated. The STI–biotin conjugates were obtained with a ratio of 1:3, and the BSA–biotin preparation was obtained with a ratio of 1:7. It is worth noting that these ratios only reflect the ratio between the number of free and occupied primary amino groups of lysine residues after conjugation. It should be noted that the STI–biotin conjugate had a higher analytical signal in this assay scheme. It is a smaller protein compared with BSA and has a smaller biotin load on the surface. However, the second conjugate, BSA–biotin, can also be used in the assays. There are about 30 available primary amino groups among the 59 accessible groups on lysine residues on the BSA surface at a pH of 7.4 [49,50].

We have shown that the synthesis conditions affect the quality of the conjugates and how even partial denaturation of the carrier protein during conjugate synthesis leads to a decrease in the conjugate’s quality [52]. Moreover, various methods for synthesizing conjugates are adapted to specific interaction conditions and depend on the nature of the conjugated agents [53]. Therefore, the remaining conjugate preparations were tested using the activated ester method but under different conditions. Two methods for synthesizing the aminophenylboronic acid conjugate with BSA and streptavidin were tested in different media: in Tris buffer, as described in our previous work [44], and in MES buffer, using one and two activators, respectively. During the preparation of the conjugates in MES, the formation of protein aggregates was observed. BSA-APBA conjugation in MES resulted in opalescence and precipitation. In contrast, no significant changes were observed visually for the STP-APBA conjugate. There was a reduction in the number of accessible amino groups in the BSA-APBA and STP-APBA conjugates (Table 1). The number of amino groups decreased more significantly when the BSA-APBA conjugate was prepared using the MES buffer and two activators. These results indirectly confirm that during the synthesis using the two tested methods, intra- and intermolecular protein covalent bonds formed between activated carboxyl groups and the primary amino groups of the protein.

**Figure 4:**
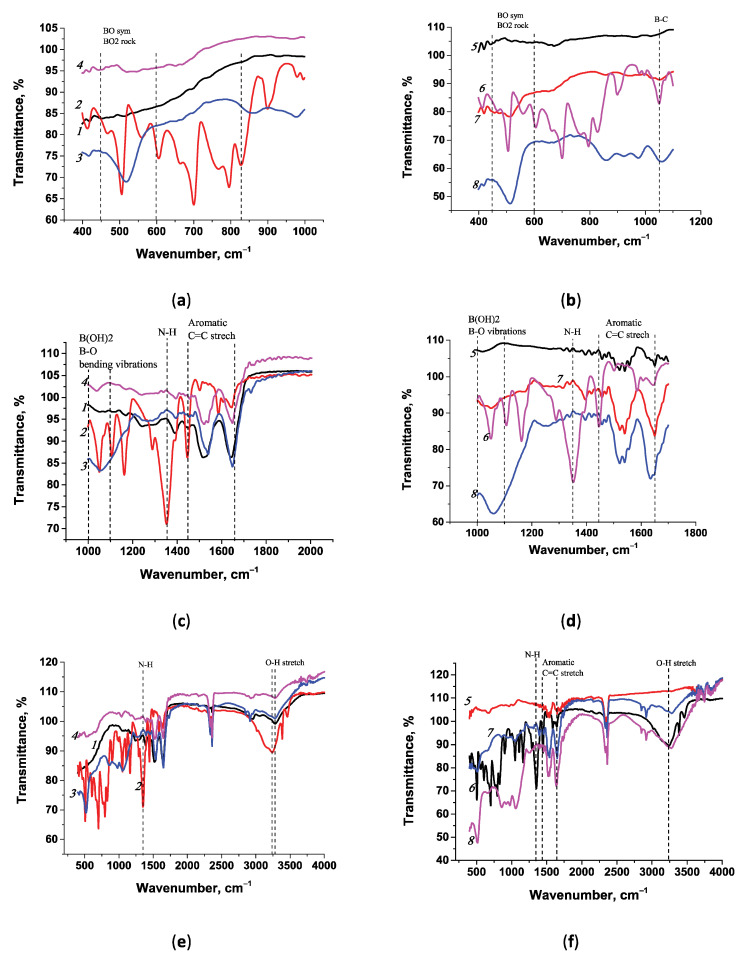
FT-IR spectra of conjugates and native components: (**a**,**c**,**e**) BSA (*1*), APBA (*2*), BSA-APBA (Tris) (*3*), and BSA-APBA (MES) (*4*); (**b**,**d**,**f**) STP (*5*), APBA (*6*), STP-APBA (Tris) (*7*), and STP-APBA (MES) (*8*). Spectra are shown for the wavenumber ranges of 400–1100 cm^−1^ (**a**,**b**), 1000–2000 cm^−1^ (**c**,**d**), and 400–4000 cm^−1^ (**e**,**f**).

The synthesized conjugates were also characterized using FT-IR spectroscopy; the results are presented in Figure 4. The spectra of the conjugates synthesized in Tris and MES buffers were compared with the spectra of the native substances (APBA) and the carrier proteins (BSA and STP). The characteristic peaks of phenylboronic acid were compared with those in [54].

Comparison of FT-IR spectra of the conjugates and native compounds showed similarities and differences in stretching vibrations. Since the presence of the main agent, phenylboronic acid, in the conjugates is of interest, a comparison of the spectra was carried out to identify the corresponding peaks. We noted the characteristic spectrum of aminophenylboronic acid, which allowed us to assess the presence of corresponding peaks in the conjugates and select the optimal reagent preparation regime. For example, the native spectrum of APBA has a characteristic N-H stretching vibration at 1340 cm^−1^, which is absent in the conjugates since the synthesis was performed using the primary amino group of APBA (Figure 4c).

Vibrational patterns characteristic of the B-O bond were examined in the spectra of all the conjugates. There are several characteristic peaks in the APBA spectrum, which can also be seen in the conjugate preparations. Assigning the symmetrical B-O stretching vibration is not straightforward, as noted by the authors of [54]. B-O vibrations located in the range of 450–580 cm^−1^ were observed in the spectra of the native APBA preparations (505 cm^−1^); they were also detected in the BSA-APBA (Tris) conjugates at 517 cm^−1^ and in STP-APBA (MES) at 514 cm^−1^ (Figure 4a,b). In the range of 450–580 cm^−1^ in the BSA-APBA (MES) conjugate spectrum, vibrations were not observed (Figure 4a), but they were present in the STP-APBA (Tris) conjugate spectrum at 512 cm^−1^ (Figure 4b).

Characteristic peaks in the spectra of the conjugates between 1080 and 1110 cm^−1^ were associated with a B-C stretching mode in arylboronic acids. These are the characteristic peaks in Figure 4b in the spectrum of APBA at 1050 cm^−1^, the STP-APBA (MES) conjugates at 1060 cm^−1^, and STP-APBA (Tris) at 1051 cm^−1^, which are absent in the native STP spectrum. The corresponding peaks were observed in the BSA-APBA (Tris) and BSA-APBA (MES) conjugate spectra (Figure 4c). O-H vibrations in the boron group were noted in the spectra of native APBA at 3235 cm^−1^, and STP-APBA (MES) and STP-APBA (Tris) at 3281 cm^−1^ (Figure 4f). Similar vibrations in the spectra of the pure substance and conjugates at 3280 cm^−1^ are shown in Figure 4e.

The spectra of the preparations allowed us to conclude that the conjugate synthesis in both Tris and MES was successful (except BSA-APBA in MES); however, the differences in peak intensity indicate possible differences in the ability to form a complex and therefore, all the reagents were tested in the subsequent experiments.

During the conjugation process in both Tris and MES, excess activators were used to bias the reaction toward the desired products when synthesizing APBA with carrier proteins (BSA and STP). At least a tenfold excess of activators relative to the amount of protein should be used according to the synthesis protocols in Hermanson’s manual [55]. For example, when synthesizing BSA-APBA in Tris, 0.32 mmol of EDC was used, and 0.16 mmol of EDC with 0.14 mmol of NHS was used to obtain BSA-APBA in MES per 0.15 μmol of BSA with 29 μmol of APBA. On the one hand, it allows for the production of conjugate preparations with a higher amount of APBA on the protein globule surface for subsequent analysis. On the other hand, it changes the behavior of the initial reagents when performing synthesis using the activated ester method under different conditions. Both APBA and Tris have primary amino groups, but they show different behaviors. Tris is an aliphatic compound, and the inductive effect is only observed in the distribution of electron density within the molecule along the σ-bonds. The primary amino group of APBA is aromatic, so Tris is more active regarding the nucleophilicity of the amino group.

The reaction conditions also greatly influence the formation of the final product. Conjugation efficiency fluctuates under reaction conditions at pHs of 6–9 because two competing processes occur: the hydrolysis of the ester group and amide formation [56]. Protonation or dissociation of different groups of the reagent occurs when the pH of the medium decreases or increases. Therefore, it is logical to assume that Tris also interacts with the activated carboxyl group to form an amide bond, thereby limiting the number of carboxyl groups in the synthesis medium. At the same time, the competition between Tris and APBA during synthesis does not exclude target interactions with the amino group of APBA, which ultimately leads to the formation of the conjugate, but with a reduced yield, as demonstrated for STP-APBA (Tris) (Figure 4, curve *7*).

The influence of the reagent concentrations, pH of the medium, protonation of amino groups, dissociation of carboxyl groups, stability of intermediate compounds, as well as the presence of competing substances in the medium, all affect the yield of conjugates using the activated ester method. At the same time, Kratzer et al. [57] showed that conjugates can be obtained in Tris using the activated ester method, provided that the appropriate synthesis recipe and quantities of reagents are used. The authors noted that the ability of Tris to block interactions with activated esters is not comparable with that of glycine or serinol.

### 3.3. Optimization of Assay Conditions

#### 3.3.1. Selection of a BSA-APBA Conjugate for Immobilization

**Figure 5:**
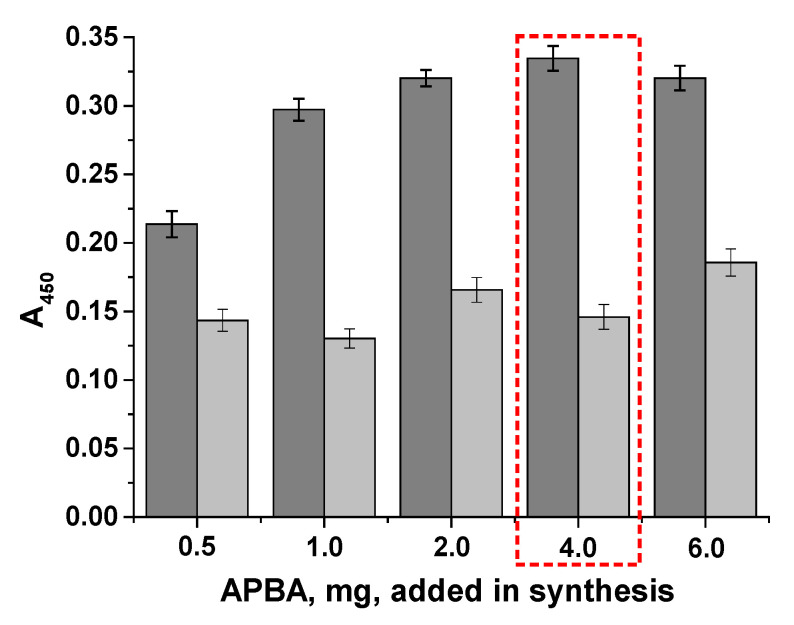
Staining intensity in the wells vs. the cell concentration and BSA-APBA conjugate. Gray bars indicate a concentration of 10^8^ cells/mL, light gray bars indicate a concentration of 10^6^ cells/mL (background staining). The selected concentration is highlighted in the red box.

A total of five conjugates were synthesized and named according to the amount of phenylboronic acid added during their synthesis, which were used for immobilization in the plate wells. We tested all the conjugates to select the optimal conjugate that would ensure cell binding and provide an adequate analytical signal (approximately 1 optical unit at 450 nm). The maximum optical density in the wells was assessed, as well as the intermediate value (at 10^6^ cells/mL) (Figure 5). The BSA-APBA-0.5 and BSA-APBA-1 conjugates produced low optical density values and a relatively high background signal, which did not increase with the amount of APBA (up to 4.0 mg) in the conjugate. At the same time, using BSA-APBA-6.0, almost the same optical signal was obtained at 10^8^ cells/mL, but there was an increase in the background signal at 10^6^ cells/mL.

Therefore, the optimal concentration of APBA 4.0 and, accordingly, the BSA-APBA-4.0 conjugate was selected at this stage.

It is worth noting that biotinylated protein conjugates (BSA and STI) were obtained using a standard biotinylation method that took into account the maximum number of diamino monocarboxylic amino acids in the protein—11 for STI and 59 for BSA. Therefore, the conjugates were obtained at a ratio of 1:15 for STI–biotin and 1:65 for BSA–biotin. These preparations were used in the analysis at the chosen concentration; no optimization was performed.

#### 3.3.2. Selecting Conditions for Interactions

**Figure 6:**
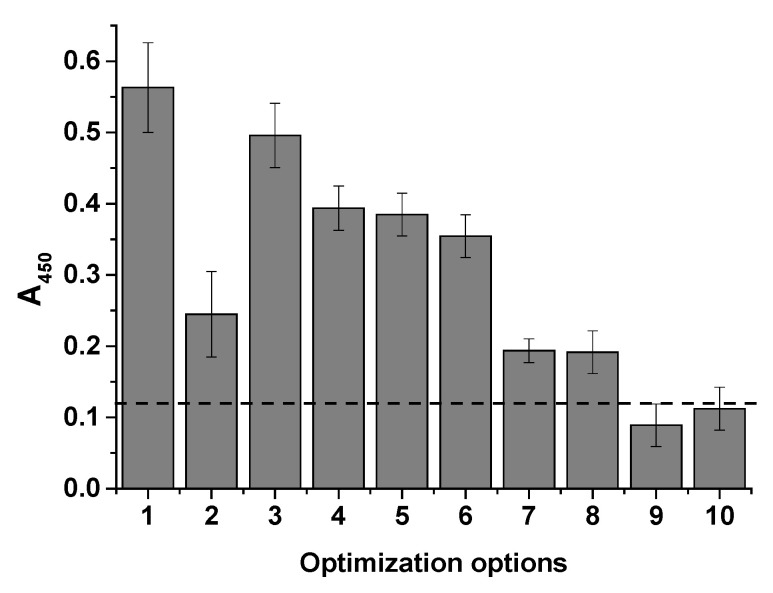
Histogram showing optical density at 450 nm vs. cell concentration (10^6^ cells/mL) under different assay conditions. *1*—STP-APBA (Tris), PBG; *2*—STP-APBA (Tris), PBT-1; *3*—STP-APBA (MES), PBG; *4*—STP-APBA (MES), PBT-1; *5*—STP-APBA (MES), PBG, blocking with 1% BSA; *6*—STP-APBA (MES), PBT-1, blocking with 1% BSA; *7*—STP-APBA (MES), PBG, blocking with 1% BSA, 0.1 mg/mL glycerin in water; *8*—STP-APBA (MES), PBG, blocking with 1% BSA, 0.1 mg/mL mannose in water; *9*—no STP-APBA; *10*—no STI–biotin. The dashed line indicates the background absorbance.

Since the system components can influence the analytical signal and interaction with cells, various assay conditions were tested. Buffer solutions (PBT-1 and PBG) and streptavidin conjugates prepared in Tris and MES were compared, and additional plate blocking was tested to reduce nonspecific signals. For direct comparison, a single *E. coli* cell concentration of 106 cells/mL was used in these experiments; the results are presented in Figure 6.

It was evident that the use of the suboptimal STP-APBA (Tris) conjugate with the introduction of detergent into the system resulted in a decrease in the analytical signal compared with the PBG buffer (columns 2 and 1 in Figure 6, respectively). This decrease is likely due to the limited amount of APBA available for binding in the conjugate, which was indicated by the FT-IR spectra. Substituting the conjugate resulted in an increase in the signal (columns 3 and 4). However, the effect of the detergent was not significant. Furthermore, blocking the microplate wells with a 1% BSA solution in PB-10 resulted in a decrease in the nonspecific signal (column 5), and further addition of Triton X-100 detergent (column 6) had virtually no effect on the analytical signal. To demonstrate the specificity of the cell interaction with the APBA in the conjugates, glycerol (column 7) and mannose (column 8) were added with the cells to the microplate wells at a concentration of 1 mg/mL, as these polyols are capable of interacting with APBA [33]. As can be seen from the last two columns, signal inhibition occurred, confirming the mechanism of interaction; these results align with those from previous studies on the mechanism of interaction with diols [26].

#### 3.3.3. Bacteria Inactivation (Storage) Method

To ensure reproducible results, the assay detects whole cells, not fragments. However, it is not always possible to analyze fresh cells. Therefore, we studied various preservation methods and storage conditions. The objective of this optimization step was to identify possible methods for storing cells without destroying them or methods for inactivating them to preserve the samples. Several options were analyzed: temperature inactivation (autoclaving for 5–20 min), addition of sodium azide to the medium, and storage as aliquots in the culture medium with 20% or 40% glycerol in a freezer. To improve cell safety, a storage method in which the cells were centrifuged and stored in PB at 4 °C was also tested.

**Figure 7:**
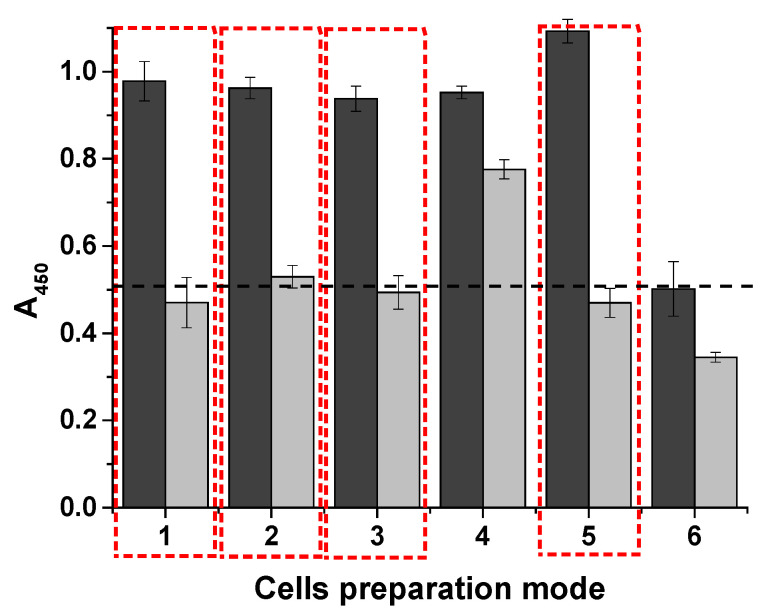
Optical density of the different cell inactivation methods (*n* = 2). Dark gray bars represent a concentration of 10^8^ cells/mL; light gray bars represent a concentration of 10^4^ cells/mL. Inactivation methods: *1*—thermal inactivation; *2*—addition of NaN_3_; *3*—20% glycerol (*v*/*v*); *4*—40% glycerol (*v*/*v*); *5*—native (fresh) cells; *6*—thawed cells after freezing. The dashed line indicates the background absorbance. The selected options are highlighted in the red boxes.

Cells prepared using the different methods were tested with the developed system and compared. The optical density in the wells was assessed at two cell concentrations: 10^8^ cells/mL and 10^4^ cells/mL (Figure 7).

It is worth noting that the native cells used in the analysis were fresh, meaning that they were grown in liquid culture medium (column 5, for comparison). An experiment was also conducted with live cells that were stored in culture medium for 24 h at 4 °C, centrifuged immediately before analysis, and re-suspended in PBS. The results were similar.

Thermoinactivation of *E. coli* was performed at 80 °C for 10 min. The thermoinactivated cells and those that were exposed to 0.008% sodium azide retained their ability to bind to the system components (columns 1 and 2). However, the addition of glycerol and storage of the cells in medium at −20 °C had different effects on their stability. The best results were obtained with 20% glycerol (column 3) compared with 40% (column 4). Of all the tested cell preparation methods, only freezing had a negative effect. Cell lysis, which occurs during freezing and thawing of samples in buffer or culture medium, negatively impacted the detection of *E. coli* (column 6). The process appears to produce fragments of varying sizes, leading to significant errors and a lack of calibration due to the limited number of binding sites for phenylboronic acid conjugates. Therefore, fresh cells or those stored in a medium supplemented with sodium azide, where the cells can maintain their structural integrity, are suitable for this assay. The number of colonies, as well as the initial concentration of living bacteria (colony-forming units) in the sample, can be calculated based on the doubling time data.

**Figure 8:**
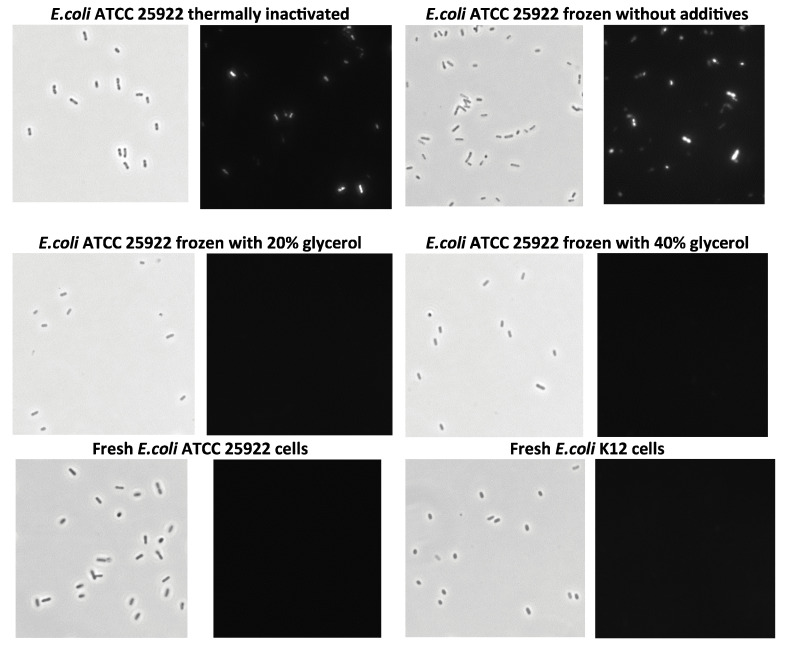
Microscopic images of *E.coli* cells stored using different preservation methods. Phase-contrast images (light pictures) and fluorescence microscopy images of bacteria (dark pictures) stained with propidium iodide (images were made at a magnification of ×1500).

A comparison of the cell inactivation and storage methods was also conducted using propidium iodide staining of bacteria and fluorescence microscopy [58] (Figure 8). Phase-contrast images show that the bacteria retained their morphology despite the various preparation methods. Therefore, a fluorescence microscope was used to assess the staining potential, as only non-viable cells can be stained with propidium iodide. It was evident that the cells ceased to be viable upon heat inactivation and freezing in buffer. However, they remained viable even when frozen at −20 °C in the presence of glycerol.

### 3.4. Calibration Curve Analysis

**Figure 9:**
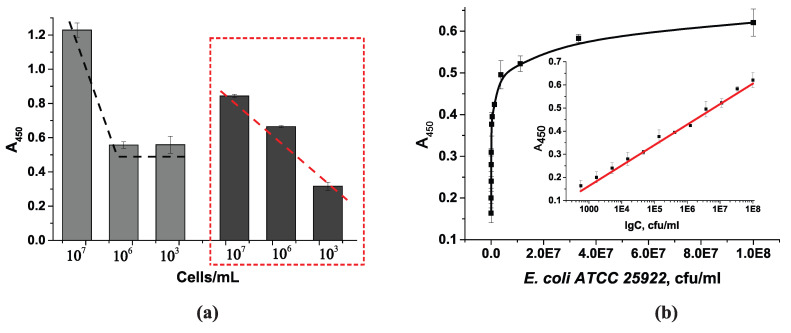
(**a**) Relationship between optical density and cell concentration with different substrate oxidation times. Gray columns represent 15 min oxidation, and dark gray columns represent 3 min oxidation. The dashed lines show the course of the curve in accordance with the optical density in the wells. (**b**) Calibration curve for *E. coli* ATCC 25922 cell detection. Insert: optical density in microplate wells vs. logarithm of number of *E. coli* ATCC 25922 cells (*n* = 8, R^2^ = 0.9922, Pearson’s r = 0.9834). LOD = 2 × 10^2^ cells/mL.

Based on preliminary interaction optimization, the parameters and conditions were selected. During optimization, the standard time for the enzymatic reaction of TMB oxidation using horseradish peroxidase as a label in the assay was used. The standard 15 min TMB oxidation time yielded slightly higher optical density values (approximately 1.2 at 450 nm with 10^7^ cells/mL), but it also increased background staining, leading to a reduction in the range of detection for cell concentrations and a detection limit of 10^6^ cells/mL for *E. coli* (Figure 9a, gray columns). Reducing the TMB oxidation time to 3 min produced low background values while maintaining a maximum optical density of approximately 0.9–1.0 at 450 nm with 10^7^ cells/mL (Figure 9a, light gray columns).

A calibration curve for *E. coli* cell counts was obtained under the selected conditions and a TMB oxidation reaction time of 3 min, as shown in Figure 9b. The range of detectable *E. coli* cell concentrations was 10^3^ to 10^8^ cells/mL. The detection limit for this system was 3 × 10^2^ cells/mL. The standard deviations across the entire operating range did not exceed 9.5%. On average, these values ranged from 0.5 to 7.4%. The average error of detection was 3.5% to 7.8% on the same day and 3.0 to 10.8% on different days; the inter-day errors (*n* = 16) did not exceed 12%.

The dependence of the optical density on the logarithm of the *E. coli* concentration is described by the equation

$$y=-0.0191+\frac{0.496+0.0191}{{1+(\frac{x}{1709559.5281})}^{-0.1569}}$$

To assess the potential influence of the sample matrix components, viable *E. coli* ATCC 25922 cells in spring water were tested. Although further cell growth in a nutrient medium will be conducted, the matrix effect was studied for a preliminary assessment of the feasibility of environmental monitoring and for planning further work outside the scope of this study. For this purpose, cell suspensions of varying concentrations were prepared and analyzed. The data were compared with a calibration curve. It was found that adding the cells in spring water to the system did not cause a shift in the results relative to the calibration curve, with the detection rates ranging from 96 to 120% (Table 2).

### 3.5. Application of Developed Technique for Detection of Pathogenic E. coli Strains

**Figure 10:**
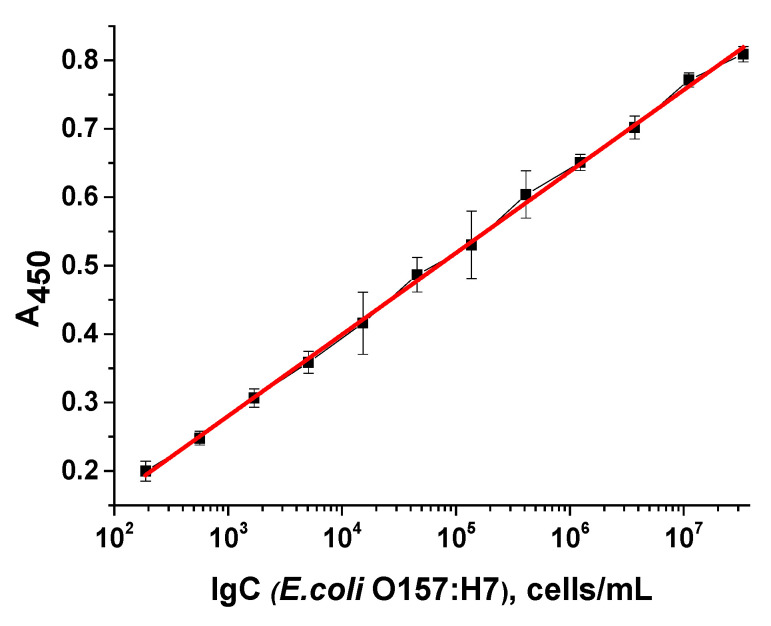
Optical density in microplate wells vs. logarithm of *E. coli* O157:H7 cell concentration (*n* = 8, R^2^ = 0.999). LOD = 4 × 10^2^ cells/mL.

The *E. coli* strain ATCC 25922 used in the previous stages is opportunistic and is used for laboratory work. Overall, regarding pathogenicity and virulence, it is not considered a dangerous microorganism, and thus, the developed assay was used to detect the toxigenic strain O157:H7, which causes foodborne infections and pathological conditions. Killed (thermally inactivated by autoclaving) cells of five clinical isolates of the pathogenic *E. coli* strain O157:H7 (OC 254, ATCC 51658, IL 38, AP 24, and OC 255) were tested at various concentrations. As shown in Figure 10, the assay showed a similar wide range of cell concentrations (10^3^ to 10^8^ cells/mL) and detection limit (4 × 10^2^ cells/mL).

Calibration curves were obtained for all isolates tested; the detection curve for a representative isolate is shown in Figure 10. In all cases, the working ranges of cell concentration were consistent, with slight variations in the detection limit—from 2 × 10^2^ to 6 × 10^2^ cells/mL. Thus, this work demonstrates the possibility of identifying both pathogenic *E. coli* cells and opportunistic microorganisms, both living and inactivated, while maintaining the integrity of the cells. The use of a pathogen inactivation method allows for cell counts to be determined outside of cleanroom conditions, which is also important for safety and the possibility of analysis outside of a microbiological cabinet.

### 3.6. Evaluation of E. coli Cells Grown on Selective Media

**Figure 11:**
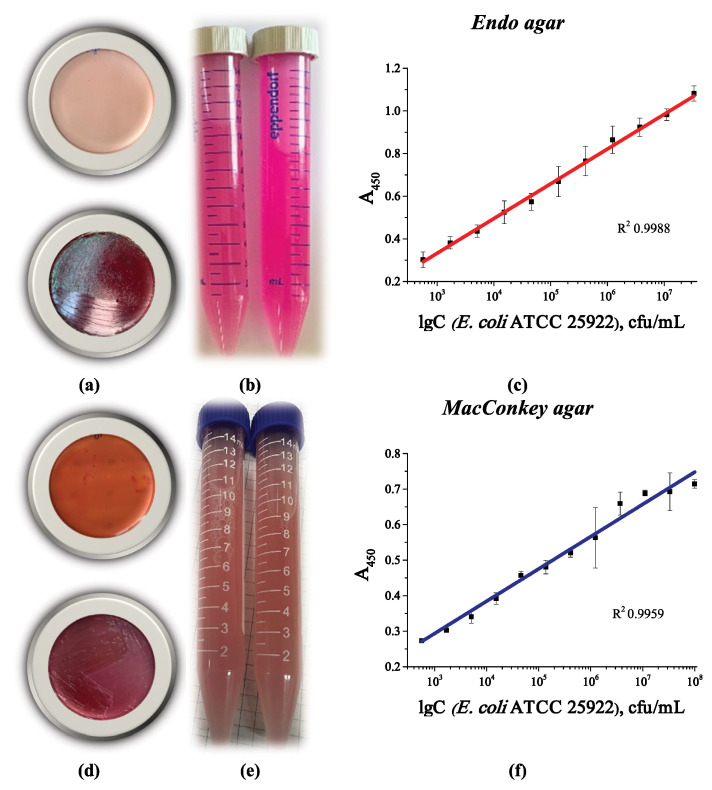
Appearance of a Petri dish with (**a**) Endo agar and (**d**) MacConkey agar before (**top**) and after (**bottom**) the growth of *E. coli* ATCC 25922 colonies; appearance of a cell suspension collected from the surface of (**b**) Endo agar and (**e**) MacConkey agar; optical density vs. logarithm of *E. coli* ATCC 25922 cell concentration after growth on (**c**) Endo agar and (**f**) MacConkey agar. The linear curves are from four replicates.

To increase the selectivity of the analysis, we propose using selective media to grow the microorganisms. Since the dye present in the medium accumulates in the cells during growth on selective media, we studied the effect of its presence on cell binding in the developed system. Endo agar and MacConkey agar media were chosen for this study. Both these media are selective for *E. coli* and some Bacteriaceae. Figure 11 shows the appearance of the Petri dishes with the corresponding selective media (Figure 11a,d), as well as the corresponding calibration curves for detecting the cells grown on these media. The main concern was whether dye accumulation (Figure 11b,e) would affect the ability of LPSs to interact with phenylboronic conjugates on the plate surface and in the medium. However, this clearly did not occur. The calibration curves were similar to those of cells grown in liquid culture medium, with similar sensitivity and working ranges (Figure 11c,f).

Thus, the developed analytical system can detect *E. coli* cells, both fresh and inactivated, that were grown in liquid nutrient medium or solid agar, including selective ones. It is possible to determine the total number of coliform bacteria after further growth on selective nutrient media. The absence of an effect of dye accumulation in the cells during growth on a selective nutrient medium was also shown.

### 3.7. Selectivity Management and Evaluation of the Capability of the Proposed Scheme in Detecting Bacterial Cells

**Figure 12:**
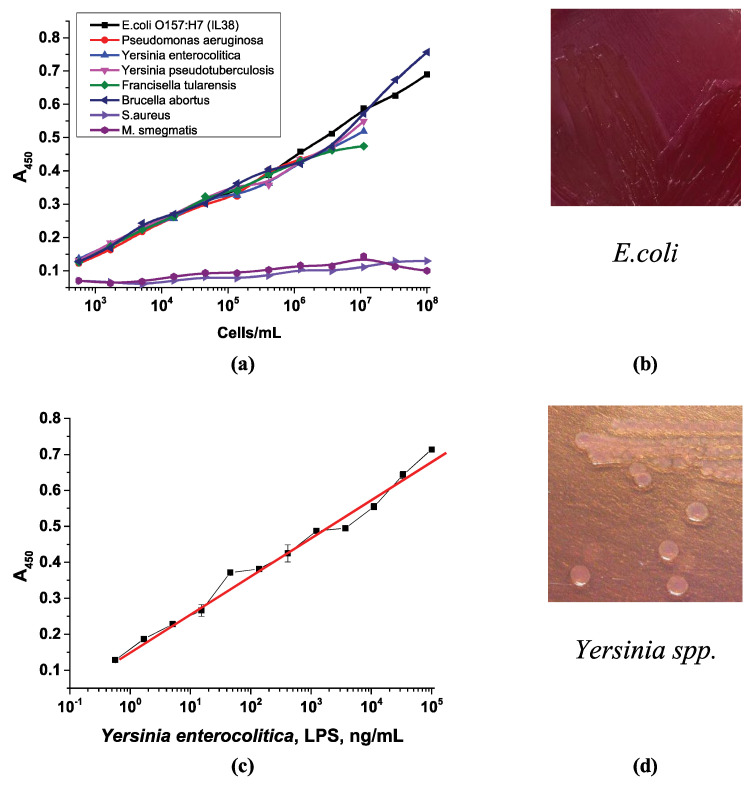
Optical density vs. logarithm of the concentration of (**a**) pure cell cultures in buffer and (**c**) LPSs of *Yersinia enterocolitica*. The appearance of (**b**) *E. coli* colonies and (**d**) *Yersinia* spp. on MacConkey agar.

In addition to testing the use of selective media, various other microorganisms were tested with the developed assay scheme: the non-pathogenic *Staphylococcus aureus* ATCC 25923 strain, a Gram-positive bacterium; *Mycobacterium smegmatis* mc^2^155 (ATCC 700084), which belongs to the phylum *Actinomycetota*; and the Gram-negative microorganisms *Salmonella enteritidis*, *Salmonella typhimurium*, *Salmonella Paratyphi A*, *Pseudomonas aeruginosa*, *Yersinia enterocolitica*, *Yersinia pseudotuberculosis*, *Francisella tularensis*, and *Brucella abortus*. All these Gram-negative microorganisms contain LPSs, but their cultivation conditions and additives in the growth media differ. They can even form different colony types on agar, especially *Pseudomonas aeruginosa* [59]. Interestingly, all the Gram-negative microorganisms (as pure cultures in buffer) could be detected by the developed system. They could interact with the APBA conjugates with the same working range as that of *E. coli* cells (Figure 12a). This is explained by the presence of LPSs in all the selected microorganisms. Since LPSs contain similar sugars in the O-antigen (rhamnose, mannose, galactose, glucose, and others), as well as cis-diol fragments, it is logical to assume that interaction with phenylboronic acid conjugates will occur. This is an advantage of the system, as it allows for the detection of total bacterial counts without discriminating between microorganisms. In contrast, neither *Staphylococcus aureus* nor *Mycobacterium smegmatis* was bound by this system since they do not have LPSs.

In addition to bacterial cells, the study utilized an existing lipopolysaccharide fragment from *Yersinia enterocolitica*. Previously, we demonstrated the negative impact of cell fragmentation, which occurs after lytic cell fragmentation during freezing and thawing (Section 3.3.3). Thus, we evaluated the interaction of an isolated LPS preparation with the developed system. Figure 12c shows the optical density in the microplate well vs. LPS concentration plot, which is similar to the curve for whole-cell detection. This is also an advantage of the developed system, as it allows for the detection of isolated LPSs as well.

However, these different strains are cultivated differently, which could be used to distinguish between them. Therefore, the assay design can be used to detect various microorganisms, but additional growth on selective nutrient media is required, which determines the selectivity of the assay. For example, MacConkey agar, the selective medium for growing *E. coli*, is only suitable for the growth of *Yersinia enterocolitica* and *Yersinia pseudotuberculosis* [60]. Therefore, these microorganisms theoretically could be detected by the developed system if MacConkey medium is chosen as the selective medium, unless the shape and appearance of the colonies are taken into account.

Another example is *Brucella abortus*, which requires Farrell’s medium or a modified *Brucella* Selective medium [61]. *Francisella tularensis*, which does not grow on simple nutrient media, requires special media containing vitamins, blood, and cysteine—Martin–Lewis’s agar, Thayer–Martin agar, chocolate agar, or cysteine heart agar [62]. *Pseudomonas aeruginosa*, the only aerobe on the list, prefers media containing carbenicillin, cetrimide agar, and Pseudomonas isolation agar [63]. *Salmonella* spp. prefers xylose–lysine deoxycholate agar, Wilson–Blair bismuth sulfite agar, enteric agar, Salmonella Shigella agar, Brilliant Green Agar, and vitamin agar [64]. However, they can grow on MacConkey medium, forming colorless or yellowish colonies on the agar surface, rather than the red ones typical of *E. coli* (Figure 12b). *Yersinia* also produces lactose-negative colonies on MacConkey agar, which are colorless (Figure 12d). Figure 12b,d demonstrate the different appearance of the colonies of the different strains and changes to the color of the MacConkey medium during their cultivation. Thus, the developed assay allows for the quantitative detection of various microorganisms, and the selectivity can be adjusted using different selective media for cell culture.

In summary, the developed system offers a number of advantages. ***First***, it is assembled entirely from conjugates obtained by chemical synthesis, eliminating the possibility of non-specific binding of reagents when replacing one bacterial strain with another. This method eliminates the need for expensive antibodies that are tailored to specific strains, as well as equipment and expensive reagents for PCR. ***Second***, it allows for the determination of the total number of coliform bacteria after growth on selective nutrient media, since we demonstrated that dye accumulation within the cells does not affect the assay results. The number of colonies, as well as the initial concentration of viable bacteria (colony-forming units) in the sample, can be calculated based on doubling time data. ***Third***, the system is versatile and enables the quantitative detection of various microorganisms. It is possible to determine the total microbial count or adjust the assay to detect a specific bacterial species by growing the bacteria on the appropriate selective medium. ***Fourth***, the system is safe and allows for the detection of both whole living cells and inactivated pathogens, as well as bacterial LPSs. The use of multiple repeating regions (LPSs) allows for the amplification of the analytical signal, increasing the sensitivity of cell detection. Thus, the assay could be used for medical diagnostics, environmental monitoring, and food safety monitoring.

## 4. Conclusions

A sandwich-type microplate assay for quantitative bacterial cell detection using low-molecular-weight compounds conjugated with carrier proteins is presented for the first time. In this assay, 3-aminophenylboronic acid conjugated to BSA and streptavidin is used to bind cells on both sides of the complex. Clasping the cells into “claws” followed by high-affinity biotin–streptavidin interaction leads to the formation of a stable complex in the wells, allowing for colorimetric detection. It was demonstrated that this interaction can be used with both native cells of an opportunistic strain and the pathogenic *E. coli* O157:H7 strain, which is of high environmental significance. A combination of microbiological, chemical, and analytical approaches resulted in a detection limit of 3 × 10^2^ CFU/mL (cells/mL) with a wide working range of 10^3^ to 10^8^ cells/mL.

The developed scheme is efficient, safe, and versatile, and can detect live cells, inactivated whole cells, and LPS alone. The use of multiple interactions due to repeating elements in the LPS structure with APBA–protein conjugates and high-affinity biotin–streptavidin interactions increases the sensitivity of cell detection and ensures a wide working range.

The versatility of this approach for pathogen detection was demonstrated using various other Gram-negative microorganisms. The selectivity of the assay for different microorganisms could be achieved through the use of specific selective media for further growth of the bacterial cells.

## Figures and Tables

**Table 1:** Fluorescence intensity and number of free amino groups in the conjugates and their native carrier proteins.

Parameter	STI	STI–Biotin	BSA
Fluorescence intensity	7915	5662	15,636
Number of free amino groups	11 [51]	8	59 (about 30 available NH_2_ groups) [50])
	**BSA-APBA-4.0 (Tris)**	**BSA-APBA-4.0 (MES)**	**BSA–Biotin**
Fluorescence intensity	12,652	3143	11,966
Number of free amino groups	48 (24 available)	12 (6 available)	46 (23 available)
	**STP**	**STP-APBA (Tris)**	**STP-APBA (MES)**
Fluorescence intensity	15,563	6621	9083
Number of free amino groups	32	14	19

**Table 2:** Results of *E. coli* cell analysis in spring water.

Cell Concentration Added	Cell Concentration Detected	Recovery, %
5 × 10^4^	6 × 10^4^	120 ± 8.8
5 × 10^5^	4.8 × 10^5^	96 ± 11.2
5 × 10^6^	5.7 × 10^4^	114 ± 12

## Data Availability

The original contributions presented in this study are included in the article. Further inquiries can be directed to the corresponding author.
